# Safety and effectiveness of intranasal dexmedetomidine together with midazolam for sedation in neonatal MRI

**DOI:** 10.1111/pan.14307

**Published:** 2021-10-12

**Authors:** Emanuela Inserra, Umberto Colella, Elisabetta Caredda, Mario Diplomatico, Simona Puzone, Sabino Moschella, Carlo Capristo, Gioacchino Tedeschi, Ferdinando Caranci, Mario Cirillo, Emanuele Miraglia del Giudice, Paolo Montaldo

**Affiliations:** ^1^ Department of Neonatal Intensive Care University of Campania Luigi Vanvitelli Naples Italy; ^2^ Critical Care Area, Anaesthesia and Postoperative Intensive Care Unit AO Ospedale dei Colli Monaldi Hospital Naples Italy; ^3^ Neonatal Intensive Care Unit AORN San Giusepe Moscati Avellino Italy; ^4^ Department of Advanced Medical and Surgical Sciences MRI Research Center SUN‐FISM University of Campania Luigi Vanvitelli Naples Italy; ^5^ Department of Precision Medicine School of Medicine "Luigi Vanvitelli" University of Campania Naples Italy; ^6^ Centre for Perinatal Neuroscience Imperial College London London UK

**Keywords:** adverse effects, dexmedetomidine, infant, magnetic resonance imaging, midazolam

Magnetic resonance imaging (MRI) has been consistently indicated as the imaging modality of choice in children and neonates. MRI is pivotal for the management and prognostication of different neonatal conditions including neonatal encephalopathy, preterm birth, and congenital heart diseases. While feed and wrap technique may be used to perform MRI scans without sedation, this is not always possible due to MRI sensitivity to motion artifacts and long duration of the procedure.

Many scans do not require contrast and the intranasal route offers an effective and relatively non‐invasive way to deliver medications. Therefore, the ideal sedation regimen should obviate the need for an intravenous access, but still enable the completion of the MRI without deep sedation and subsequent complications. Dexmedetomidine is a highly selective α2 adrenergic receptor agonist, which induces dose‐dependent sedation, anxiolysis, and sympatholysis with respiratory drive preservation. Although it has been increasingly used in pediatric sedation for diagnostic imaging, little it is known on its safety and efficacy for procedural sedation in newborns.

Recently, the Pediatric Sedation Research Consortium reported the data regarding the safety and efficacy of intranasal dexmedetomidine for sedated MRI examinations in children.^1^ The authors found 224 sedation encounters, 216 (96.4%) of which, also received intranasal midazolam. In all these cases, no major nor minor adverse events were reported.

However, no neonates were included in the study.

Bua et al.^2^ assessed the use of dexmedetomidine as midazolam‐sparing drug to achieve sedation for MRI in 53 preterm neonates scanned at term equivalent age. The authors reported a good safety profile of dexmedetomidine without the need of any medical intervention during the MRI scans. However, in almost half of the infants (49%), one or more doses of midazolam were needed as adjunct to complete the procedure. Moreover, no data on the duration of the MRI scans, degree of sedation, or the quality of images were provided.

The primary aim of our study was to assess safety and effectiveness of the combination of intranasal dexmedetomidine and midazolam as sedation for neonatal MRI compared with intranasal dexmedetomidine or intranasal midazolam alone.

We prospectively studied 199 neonates scanned at the MRI Research Centers of the University of Campania “Luigi Vanvitelli” and AORN San Giusepe Moscati, Italy, from September 2019 to July 2021. The first one‐hundred‐one infants received a single dose of intranasal dexmedetomidine 3 mcg/kg 20 min before scheduled MRI and 10 min after dexmedetomidine administration, intranasal midazolam (0.2 mg/kg). The following 98 infants received a single dose of intranasal midazolam (0.2 mg/kg) 10 min before the MRI scan. A historical group of 78 infants born in the previous 2 years and who received intranasal dexmedetomidine (3 mcg/kg) as first‐line drug was used as third comparison group. No changes took place in the MRI protocol during the study period. In all the three groups, intranasal midazolam (0.2 mg/kg) was administered as rescue sedation. All the neonates were continuously monitored from the time of sedation to full waking. Intranasal route was achieved by using a mucosal atomization device.

In all the infants, we placed peripheral venous access before the scan and adjusted feeding times (last feed 2 h before the scan), minimized noise with double‐layered hearing protections and light with eye shields during all the scans. The degree of sedation before and after drugs administration was assessed by using N‐Pass Score. A single researcher (PM) scored image quality from 1 (poorest) to 5 (best) for T1 and T2 images based on gray‐white matter differentiation, deep nuclei organization, and the absence of motion artifact, on axial images. An overall quality score of 1–5 was given based on both T1 and T2 scans.

Adverse effects of sedation were defined as cough, bradycardias (heart rate <100 beats per minute), desaturations (oxygen saturation <90% lasting >10 s),^3^ and apneas (cessation of respiration for >20 s). We recorded if any medical intervention such as head repositioning or stimulation was needed.

Table 1 shows demographic, clinical features and sedation performance. The median time to achieve sedation was shorter in the midazolam group. However, sedation failure rate was lower, and the median quality score of the scans was higher in the dexmedetomidine plus midazolam group. The regression model adjusted for gestational age at birth, sex, weight, and gestational age at MRI, showed that dexmedetomidine and midazolam alone groups had higher odds of sedation failure (OR 6.6, 95% CI 2.8–15.1 and OR 11.6, 95% CI 5.3–25.3, respectively, *p* < .0001). Desaturations were not self‐limiting in six cases (two in the dexmedetomidine plus midazolam and four in the midazolam alone groups) but responded promptly to supplemental oxygen. No other interventions or assistance was required for any participants.

**TABLE 1:** Demographic and clinical characteristics of the patients

	Dexmedetomidine plus Midazolam (*n* = 101)	Dexmedetomidine alone (*n* = 78)	Midazolam alone (*n* = 98)
Sex (male)	59 (58)	41 (53)	56 (57)
Gestational age at birth (weeks)	36.4 (33.6–38.8)	37.3 (35.4–39)	37.7 (35.6–39.7)
Gestational age at scan (weeks)	39.3 (38.1–40.4)	40.2 (38–41)	38.6 (37.7–39.7)
Chronological age of term infants	10 (7–23)	14 (8–23)	12.5 (7–36)
Birthweight (kg)	3.2 (2.4–3.5)	3.08 (2.49–3.32)	3.1 (2.6–3.4)
Weight at scan (kg)	3.5 (3.2–3.7)	3.2 (2.6–3.3)	3.7 (3.1–3.9)
Duration MRI scans, minutes	59 (49–67)	54 (36.5–69)	56 (39–78)
Dexmedetomidine, mcg/kg	3	3	‐
Number of doses of midazolam (0.2 mg/kg)			
0	0	46 (59)	0
1	92 (91)	29 (37)	34 (35)
2	8 (8)	2 (3)	60 (61)
3	1 (1)	1 (1)	4 (4)
N‐Pass Score before sedation	0 (0–0)	0 (0–0)	0 (0–0)
N‐Pass Score after sedation	−4 (−4 − −4)	−4 (−3 − −4)	−4 (−3 − −4)
Indications for brain MRI			
Hypoxic‐ischemic encephalopathy	67 (66)	56 (72)	64 (65.3)
Preterm at term follow‐up	16 (16)	8 (10.2)	13 (13.3)
Seizures	8 (8)	8 (10.2)	10 (10.2)
Persistent pulmonary hypertension of the newborn	4 (4)	1 (1.3)	3 (3.1)
Hypoglycaemia	1 (1)	1 (1.3)	2 (2)
Stroke	5 (5)	4 (5)	6 (6.1)
Quality score	5 (4–5)	4 (3–4)	4 (3.25–4)
Time to achieve sedation (minutes)	15.2 (13–18)	19 (15–22)	9 (8–11.5)
Sedation failure^a^	12 (12)	32 (41)	64 (65)
Adverse effects			
Self‐correcting bradycardia	6 (6)	8 (10)	2 (2)
Transient oxygen desaturations	3 (3)	2 (3)	7 (7)
Need for supplemental oxygen	2 (2)	0	4 (4)

Note

Data are presented as *n* (%) for categorical variables and median (inter‐quartile range) for continuous variables.

^a^ Sedation failure was considered if the MRI scan had to be interrupted because of motion and/or awaking of the neonate or a further dose of either the same or another sedative agent was required.

Our data show that the use of intranasal dexmedetomidine together with midazolam provided an adequate sedation even in scans of long duration, with low risks of significant adverse effects and high‐quality images. Although dexmedetomidine offers favorable sedation profile with minimal respiratory depressant effects, it has slow onset of action when compared to other sedatives. The combination of dexmedetomidine and midazolam increased the efficacy of procedural sedation in neonates and can be considered an option in case of MRI scans of long duration.

In conclusion, intranasal dexmedetomidine as an adjunct to intranasal midazolam offers relatively efficient and effective sedation with minimal adverse effects for neonatal MRI scans when compared to intranasal dexmedetomidine or midazolam alone. Intranasal route offers a viable alternative for neonates who do not have an indwelling intravenous access at the time of their scan.

## CONFLICT OF INTEREST

None.

## ETHICAL APPROVAL

The study protocol was approved by the University of Campania “Luigi Vanvitelli” ethics committee.

## ACKNOWLEDGEMENT

Open Access Funding provided by Universita degli Studi della Campania Luigi Vanvitelli within the CRUI‐CARE Agreement. [Correction added on 20 May 2022, after first online publication: CSAL funding statement has been added.]

## Data Availability

Data are available upon reasonable request and once all the different substudies have been published.
